# Hard Tissue Preservation in Minimally Invasive Mandibular Third Molar Surgery Using* In Situ* Hardening TCP Bone Filler

**DOI:** 10.1155/2018/5274754

**Published:** 2018-11-11

**Authors:** Wilfried Engelke, Marcio Lazzarini, Víctor Beltrán

**Affiliations:** ^1^Georg-August-University Hospital, Department Oral and Maxillofacial Surgery, Göttingen, Germany; ^2^Max Planck Institute of Experimental Medicine, Department of Molecular Biology of Neuronal Signals, Göttingen, Germany; ^3^Universidad de La Frontera, Dental School, Clinical Investigation and Dental Innovation Center (CIDIC), Temuco, Chile

## Abstract

**Background:**

Maintenance of hard tissue in the case of impacted third molars (M3M) with close relationship to the mandibular canal is still a surgical challenge which may be overcome using the inward fragmentation technique.

**Methods:**

A consecutive case series of 12 patients required the extraction of 13 impacted M3M with a close relationship to the inferior alveolar nerve (IAN). Via occlusal miniflaps, M3M were exposed occlusal under endoscopic vision and removed by inward fragmentation. All patients received socket preservation with resorbable in situ hardening TCP particles to reduce the risk of pocket formation at the second molar.

**Results:**

All 13 sites healed uneventfully. Bone height was assessed using CBCT cross-sectional reformats pre- and 3 months postoperatively. The bone height was reduced by 1.54 mm lingual (SD 0.88), 2.91 mm central (SD 0.93), and 2.08 mm buccal (SD 1.09). Differences were significant at a 0.05% level. No tissue invagination at the extraction sites was observed.

**Conclusions:**

Major bone defects can be avoided safely using inward fragmentation surgery. The self-hardening bone filler appears to enhance the mineralization of the intrabony defect.

## 1. Introduction

The extraction of mandibular third molars (M3M) is one of the most frequent interventions in oral surgery. Despite a routine intervention, M3M surgery may cause a wide range of intra- and postoperative complications from mild discomfort and edema to inflammatory complications up to major bone defects, which in critical cases may lead to mandibular fractures [1]. Furthermore, periodontal defects with exposure of the adjacent second molar root surface may occur as side effects of the osteotomy [2]. A main complication of M3M surgery is a sensory disturbance of the inferior alveolar nerve; the incidence varies between 1.3% and 5.3% [3]. Concerning the intra- and early postoperative complications, increased operating time and advancing age are associated with more postoperative morbidity, particularly in distoangular and horizontal impaction types [4]. Additionally, the experience of the surgeon appears to play a major role in the incidence of postoperative complications [5]. There is some evidence that an incision technique which avoids an L-shaped classical incision may allow a less traumatic surgery and a lower rate of postoperative symptoms [6]. Recently, the use of low invasive surgical strategies to reduce morbidity is being discussed widely in order to reduce intra- and postoperative complications [7]. It is obvious that optimization of M3M surgery should include the preservation of as much hard and soft tissue as possible. Since bone exposure, even without bone removal or extraction, leads to bone resorption [8, 9], modern surgery should use a minimally invasive access for hard tissue preservation, which in turn requires a precise visualization of the surgical site [10]. For that purpose, support endoscopy (SE) has been used recently to visualize the extraction site of partially impacted M3M [11, 12]; endoscopic assistance also was reported in a case of an ectopic molar in the subcondylar region [13]. SE has been shown as a key technique not only for third molars but also for direct observation of a variety of alveolar structures [14]. SE contributes to obtain a good visualization of the surgical site in case of complex M3M surgery, root fractures, and nerve exposure and allowed to develop the inward fragmentation technique (IFT) with an occlusal miniflap approach [12]. First results of IFT showed that the anatomical integrity of the site could be maintained in highly complex cases with a buccal bone loss of only 1 mm and a low complication rate; however, the measurements were based on panoramic x-rays and intraoperative probing. Meanwhile, cone-beam computed tomography (CBCT) has been used for treatment planning as well as for control purposes [15] and can be used to obtain data for comparative 3D pre- and postoperative hard tissue measurement.

Although an occlusal approach may solve the problem of buccal bone loss, investigations have shown for M3M surgery [16] that the removal of the third molar often does not resolve the periodontal problem encountered distal of the second mandibular molar [17]. Partially exposed M3M frequently show a postoperative tendency to a central alveolar void space, which may cause food retention and serve as a potential source of infection. Furthermore, the loss of bone can lead to formation of periodontal pockets at the distal aspect of the second molar [18–23].

The aim of the present report was focused on the hard tissue changes of the alveolar bone following the removal of M3M with IFT in combination with the use of an in situ hardening bone filler for socket preservation. Changes of the adjacent buccal and lingual bone walls should be measured during routine IFT surgery of complex impacted M3M and the result of hard tissue formation following socket preservation should be evaluated.

We expect the application of a self-hardening TCP material in combination with the minimally invasive surgical technique should help to minimize loss of the crestal volume and therefore minimize an invagination of tissue in the crestal area and formation of formation of pockets at the second molar.

## 2. Patients and Methods

A consecutive case series of 12 patients (5 men; 7 women) with mean age of 23.5 years (min 20; max 33) were included in this case series. Patients had been referred to the Center of Oral Microsurgery at the Faculty of Dentistry of the Universidad de la Frontera (UFRO), Temuco, Chile and to the Department of Maxillofacial Surgery, Universitätsmedizin Göttingen (UMG) in 2014-2015. The study was approved by the UMG Ethics Committee (Decision 10/02/14) and UFRO Ethics Committee (Decision 011_17).

In the present case series, completely or partially impacted mandibular third molars with the absence of acute inflammatory symptoms were included, and erupted molars were excluded. All M3M exhibited a close relationship to the mandibular canal, with vertical position or mesial angulation. Exclusion criteria were missing pre- or postoperative CBCT data and general medical risks. The patients presented with the following indications: chronic pericoronitis, pericoronal cysts, orthodontic reasons (lack of space). Eight of thirteen M3M showed direct contact with the mandibular canal, with the remaining sites presenting a close relationship with a distance from the root tip to the mandibular canal below 2 mm. Thus, all cases belonged to a high-risk group for postoperative neurosensory disturbances. All patients signed an informed consent concerning pre- and postoperative diagnostics, the surgical technique to be applied, the use of biomaterials, and the risks of complications as well as the use of CBCT in pre- and postoperative surgery.

For planning of the microsurgical extraction, a CBCT scan was taken (*PAX Zenith 3D cone* beam tomograph, Vatech Co., Hwasung, Korea). The scanning parameters were 100-105 kV, 24 s, and 5.0-5.6 mA, the voxel size was 0.2 mm, and field of view was 16 cm×14 cm. CBCT images were processed and observed with the Ez3D Plus Professional K software (Vatech Co., Hwasung, Korea). CBCT cross-sectional multiple reformats were obtained to detect tooth position, complexity of root anatomy, relation to the mandibular canal, and the adjacent structures.

### 2.1. Surgical Procedure

Surgery was performed under local anesthesia (4% articaine with 1:100,000 epinephrine). The surgeon worked in a 12 o'clock position observing the site on a video screen via a Storz Hopkins support endoscope (30 view angle, 2.7 mm or 4 mm diameter, Karl Storz, Tuttlingen, Germany). The support endoscope was placed posterior to the surgical site.

**Figure 1:**
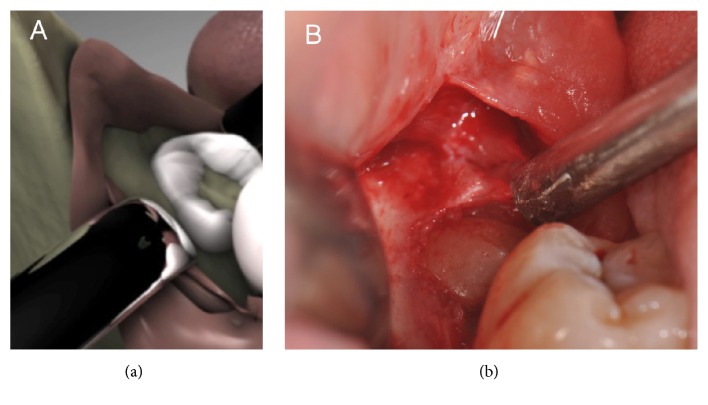
The occlusal exposure. Schematic diagram (a) and intraoperative view (b).

**Figure 2:**
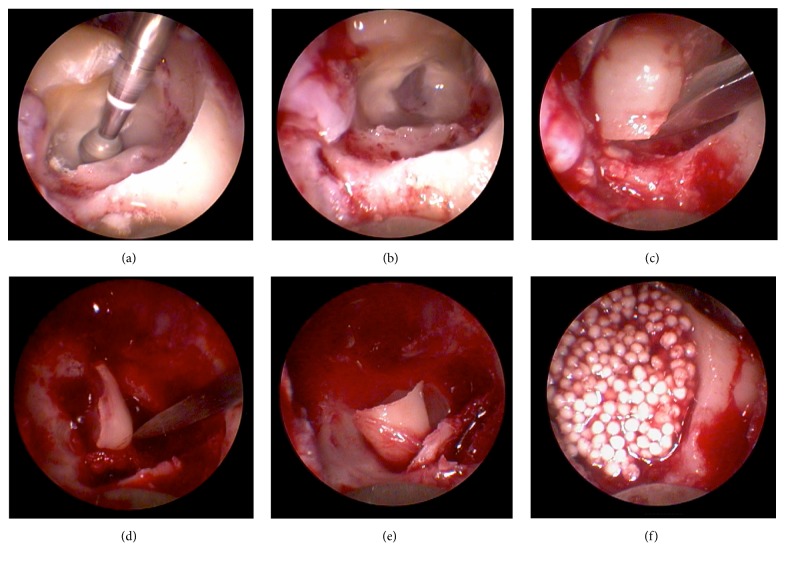
Right side M3M surgery with socket preservation (distomesial endoscopic perspective). (a) Central cavity formed. (b) Separation competed. (c) Crown fragment mobilized. (d) Mesial root mobilized. (e) Distal root mobilized. (f) Socket preservation using in situ hardening easy-graft material.

A sulcus incision was performed near the mesiobuccal edge of the second molar to its distal surface. The incision line continued sagittally towards the mandibular ramus along the extension of the M3M (see Figure 1). Soft tissue reflection was carried out over the crest only to allow the insertion of the support endoscope at the distal aspect of the site; no reflection of the periosteum was made on the lateral and lingual aspects of the site. Crestal exposure of the M3M was restricted to the occlusal aspect only, regardless of the angulation and degree of impaction of the tooth. Trepanation of the M3M was performed in order to provide access to the pulp (see Figure 2(a)); the trepanation was oriented in a transverse direction to create an internal space-making cavity. The transverse cut was performed with Lindemann as well as carbide round burs in the buccal and central parts of the crown with the exception of the lingual aspect to expose the furcation area. Lingual crown separation was carried out separately under endoscopic vision using diamond round burs. After full exposure of the furcation area, the crown was removed by inward fracturing; the roots were mobilized and removed under direct support of endoscopic vision [12].

Once the cavity was cleaned with sterile saline solution and checked, it was filled with a self-hardening alloplastic bone substitute (GUIDOR* easy-graft *CLASSIC, Sunstar Suisse SA, Etoy, Switzerland) (Figure 2(f)). In case of an exposed inferior alveolar nerve, a collagen sponge (Parasorb, Resorba Medical Nürnberg, Germany) was placed in the apical part of the alveolus. Wound closure was performed with interrupted 3-0 silk sutures. All patients received paracetamol 500 mg 4 times daily; additionally, an antibiotic treatment (amoxicillin 750 mg, 3 times daily) was administered for 4 days. Clinical follow-up visits were scheduled on days 2 and 7 after surgery; the sutures were removed at day 7.

### 2.2. Statistical Analysis

The statistical analysis was performed using the statistical software program SPSS version 23 (IBM Corp, USA). Descriptive statistics were used to indicate the mean, minimum and maximum values of the clinical parameters. Differences in the pre- and postoperative dimensional changes of the alveolar ridge were tested by the paired t-test for parametric samples. The score values of swelling and pain comparing day 2 versus day 7 were tested using the nonparametric Wilcoxon test, and the level of significance was set at P < 0.05.

## 3. Results

### 3.1. Evaluation

**Figure 3:**
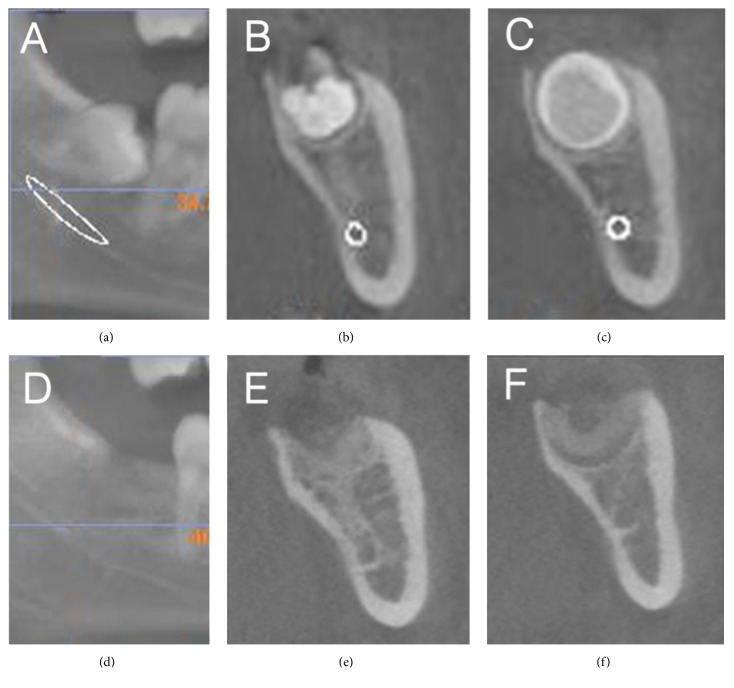
CBCT before (a, b, c) and 3 months after (d, e, f) M3M removal and socket preservation with beta-TCP bone filling material. Sagittal representation (a, d) and cross-sectional reformats at 3 and 6 mm distance to the second molar (b, e) and (c, f). The cross sections (c) and (f) represent the center of the socket and were used for bone height measurements.

**Figure 4:**
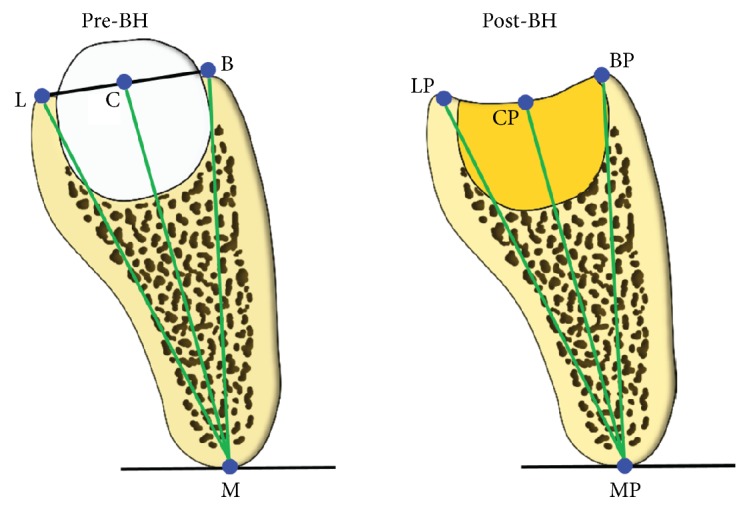
Schematic representation of CBCT measurement. Left: preoperative (Pre-BH) measurements of buccal (M-B), central (M-C), and lingual (M-L) bone height before surgery. C is defined as center of the connecting line L-B. Right: postoperative (Post-BH) measurement of buccal (MP-BP), central (MP-CP), and lingual (MP-BL) bone height. CP is defined as the center of the hard tissue contour.

Primary outcome parameters were preoperative bone height (Pre-BH) and postoperative bone height (Post-BH) 3 months following extraction, swelling, and pain level at 2 days, pain duration, and postoperative complications. The Pre-BH and Post-BH were assessed from CBCT cross-sectional reformats perpendicular to the occlusal plane 6 mm distal from the distal contour of the second mandibular molar M2M (see Figure 3). BH was measured as the distance between the mandibular base (M) and the most cranial points of the buccal (B), central (C), and lingual (L) occlusal surface of the M3M site (see Figure 4). Clinical controls took place at 2 and 7 days after surgery. At 2 days, the pain level was determined on a 10 cm visual analog scale (VAS), and the degree of swelling was ranked on a scale from 0 to 3: 0: no swelling; 1: light swelling (just visible); 2: moderate (local) swelling; and 3: severe (extended) swelling. One year following surgery, the patient's files were reviewed for postoperative complications and for quantitative evaluation of the CBCT files. To minimize the risk of bias, a surgeon who had not operated on the patients conducted the postoperative examinations.

Based on the review of the patient files, intra- and postoperative complications like bleeding, nerve lesions, mandibular fracture, or accidental displacement of root fragments were not found. Some discharge of TCP particles was reported in 5 of 12 cases during the first week of follow-up. A mild level of pain and swelling was observed (see Table 1), which decreased significantly during the first week of observation (Wilcoxon paired t-test; p<0.05).

### 3.2. Bone Height Measurement

The CBCT measurements of mandibular bone height at the M3M sites obtained from 12 patients with 13 sockets preoperatively and 3 months postoperatively are displayed in Table 2.

The lingual bone height ML decreased by 1.54 mm, SD 0.88 mm (p=0.019). The central bone height MC decreased by by 2.91 mm, SD 0.93 mm (p=0.014) at 6 mm. The buccal bone height MB was reduced by 2.08 mm, SD 1.09 mm (p=0.049). The preoperative central bone height was measured as the center point of the connection line between the buccal and lingual bone margins. Compared to this level, the central bone loss was 2.91 mm (p=0.014) (see Table 2).

## 4. Discussion

The present evaluation was part of an ongoing quality control study of the endoscopic inward fragmentation technique described earlier [12]. Using CBCT in selected cases instead of panoramic x-rays with intraoperative probing, the exact pre- and postoperative bone dimension can be assessed in M3M sites with great accuracy in preselected cross-sectional reformats. To the knowledge of the authors, the present report is the first case series which provides precise pre- and postoperative anatomical measurements in M3M sites. Compared to the data presented in 2014, the measurement was taken on a transverse cross-sectional plane pre- and 3 months postoperatively. This perspective makes it possible to differentiate structures, which in conventional panoramic x-rays cannot be discerned due to superimposition.

The buccal bone margin was measured in the previous study [12] as the distance of the apical point of the alveolus and the buccal bone contour along the longitudinal axis of the inferior third molar. Intraoperative control was obtained by probing along the axis of the tooth to the buccal side with reference to the most apical extension of the alveolus. This measurement has some shortcomings due to different measuring devices, limited reading accuracy, and possible optical distortion during the intraoperative measurement. These shortcomings could be overcome in the present study using transversally oriented reformats at a well-defined distance to the second molar at both measurement time points.

The data show that the buccal bone loss in the present case series (2.08 mm) was 1.2 mm higher than in the previous study (0.8 mm). This may be due to the following: (1) measurement errors using a periodontal probe with a reading accuracy of 0.5 mm, (2) limited reading accuracy of panoramic x-rays which cannot fully discern buccal and lingual structures, (3) different measurement times: postoperative bone resorption following a marginal exposure of the M3M site could be a factor aggravating the marginal bone loss and would not be measured intraoperatively, and (4) extended osteotomy compared to the previous case series. A review of the cases revealed that the number of fully included 3rd molars was similar in both case series; however, the amount of osteotomy may have varied, because a recently trained surgeon with less experience using IFT was involved in the present procedure.

Compared to the exact measurement of buccal and lingual bone margins, the central site measurement has some methodical shortcomings. Due to the presence of the tooth preoperatively, only a virtual center of the M3M alveolar occlusal limit can be assessed at that time. This virtual delineation was calculated on the basis of the buccal and lingual alveolar margins. Complete healing would imply that the regenerated hard tissue filled the complete alveolus up to the virtual preoperative level. The measurements, however, show that the center of the socket does not reach this level but exhibits a level 2.9 mm below the original virtual alveolar surface; nevertheless, there is only a 1.27 mm difference between the hard tissue level in the center and the postoperative buccal plate level. This value indicates that using a commonly practiced socket preservation technique with an* in situ* hardening bone filler, the clinician may encounter only a slight concavity, i.e., a hard tissue level difference of less than 1.5 mm, which clearly indicates favorable conditions for long-term stability of the adjacent periodontal tissues. A central socket defect, which might be the cause of postoperative food retention and secondary healing, was not observed, which may be a positive effect of using the* in situ* hardening bone filler.

The lingual site measurement exhibited a bone loss of 1.44 mm. A preoperative measurement on a panoramic x-ray is difficult; therefore, no preoperative data were available in the previous report. The actual CBCT data show that between the buccal and lingual alveolar margins there is only a small difference of 0.55 mm more bone loss at the buccal site. This difference can be easily explained by the surgical approach, where there is always a tendency to remove more tissue on the buccal side in the working direction of the instruments. Additional postoperative bone resorption due to the crestal marginal exposure of the bone also may contribute to explaining the different levels. However, the data show that the maintenance of the buccal plate in the previous and the present report does not imply a “compensatory” lingual bone loss, so that a preservation of the M3M site is obtained both buccally and lingually to a widely similar degree.

The clinical data give evidence that swelling and pain scores do show relatively low values as reported for flapless surgery by Kim* et al.* (2011) [16] and Engelke* et al.* (2014) [12].

Although the results indicate a significant reduction in bone height, the data show that more 90 percent of the total height at the buccal and the lingual site are maintained using IFT. This indicates a clinically favorable result concerning the vertical dimension and shows that a spontaneous fracture risk following surgery can practically be excluded. Furthermore, it appears that the alveolar socket defect after a third molar extraction is transformed satisfactorily into mineralized tissue and that the bone architecture of the marginal crestal frame at 3 months is supported.

A recent study [24] showed that CBCT is a valuable diagnostic adjunct for identifying an increased risk of IAN injury but that the use of CBCT does not translate into a reduction of IAN injury or other postoperative complications after removal of the complete mandibular third molar. The present results confirm earlier observations that the use of SE for intraoperative visualization of inward fragmentation represents an efficient tool which can be applied successfully to avoid complications in complex cases. The use of surgical guides planned with 3D data sets may help in future to identify precisely the furcation area and its relation to the IAN during surgery. Meanwhile, the intraoperative visualization and the use of atraumatic instruments appear to be technically adequate for operating in close distance to the IAN to avoid functional disturbances even in the case of nerve exposure. Future studies may reveal whether alternative low invasive strategies such as pericoronary osteotomy [25] with secondary removal after spontaneous eruption, a two-stage technique to assist the eruption [26] or an orthodontic approach to reduce the risk of an IAN lesion may lead to similar or better results.

Although there was a critical preoperative location of the M3M close to the IAN, the data presented here show that the occlusal approach was sufficient to detach the M3M from its proximity to the IAN without lateral root exposure. Thus it can be shown that M3M removal in critical cases with an IAN lesion risk does not require a large vertical reduction of the buccal cortical plate to obtain adequate vision. In contrast, the occlusal approach may be seen as a shift in paradigms for M3M surgery, provided that adequate surgical tools are used to perform inward fragmentation. Coronectomy [27] as an alternative procedure also endeavors to avoid alveolar nerve injury completely. However, an eruption of the retained root(s) was observed in 23.8% of the patients and insufficient growth of new bone in the alveolar defect was present; in 11.3% a second surgical procedure was required to remove the root remnants. In conclusion, the IFT appears to be an adequately safe procedure with practically no need for secondary surgery and a good recovery of the hard tissue if used in combination with in situ hardening bone filler.

The values show that an increased fracture risk as observed in partially erupted M3M for mandibular angle fractures [28] may be avoided using the technique at least.

The resting difference of bone height appears to be a result of pericoronary exposure of the occlusal aspect of the M3M and may also be a result of occlusal bone resorption during the postoperative healing process. Assuming that a conventional reduction of the buccal plate to expose the furcation zone of an M3M would require at least a reduction of 6 to 10 mm, the bone loss during surgery appears comparatively low and confirms the feasibility of previous anecdotal reports on an occlusal approach to M3M.

Mobilizing the crown fragments in an inward direction requires a space of about 5 mm mesiodistal extension and with complete transverse reduction of the enamel to the borders of the socket. This preparation of the cavity is more time consuming than a simple separation, but less time consuming than a piezosurgical extraction [29]. An important advantage is the opportunity to mobilize convergent roots in an inward direction. This frequent anatomical situation of curved roots with an opposite curvature of mesial and distal roots may be solved easily and allows a simple and rapid mobilization of any root anomaly after having completed the central cavity. It should be stressed that only a complete transversally directed reduction allows the inward mobilization; therefore, in the lingual zone the removal of enamel and root dentine must be performed using diamond round burs which in case of touching the lingual periosteum does not represent a risk of soft tissue damage. It may also be combined with an internal microosteotomy using piezosurgical tools.

The depth of the resulting alveolus from removal of the M3M depends on the extension of the tooth but may easily reach up to 20 mm depth, which in turn and particularly during a flapless approach may result in postoperative formation of periodontal pockets. In the case series presented here, all patients received an in situ hardening bone filler for socket preservation. Concerning the periodontal regenerative capacity, Tabrizi* et al.* (2014) [30] found that reconstructive procedures such as DFDBA did not offer predictable benefits compared with a nontreatment group in patients younger than 30 years. Kumar* et al.* (2014) [31] reported that the application of PRF lessens the severity of immediate postoperative sequelae, decreases pocket depth, and hastens bone formation. Cortell-Ballester* et al.* (2015) [32], in an RCT comprising 30 patients each, found after mesioangular or horizontally impacted 3M that the use of resorbable membranes stimulated bone regeneration, improved the attachment level and resulted in faster recovery. They recommended the placement of resorbable membranes to prevent periodontal defects. Another study described the use of HA particles and PRP in comparison with control sites: an improvement in wound healing and increase in bone density were observed in the study group; however, the parameters were not significant [33]. Barbato* et al. *(2015) [34] found in a systematic review that periodontal healing following extraction of M3M may benefit from GTR-based procedures compared to nongrafting procedures; however, an overall low quality of evidence was found.

Postoperative inflammatory complications like alveolitis may occur even following low traumatic piezosurgery [29]. The present case series did not show any purulent discharge from the sockets, which may be an effect of the socket preservation.

CBCT was used successfully in our patients for displaying the mandibular canal. As Shokri* et al.* (2014) [35] pointed out, the areas with the most visibility of the IAN in CBCT`s on the right and left sides were the second and third molar regions, respectively. This favorably provides sufficient preoperative information about the site or risk, which can be identified intraoperatively with the endoscopic imaging technology to ensure intact anatomical conditions. In the future, similarly to flapless implant surgery [36], a 3D surgical splint-guided preparation could be applied to obtain precise access to the M3M furcation area with a well-defined distance to the neurovascular bundle.

There is no evidence at present that variation of surgical techniques of a transposed versus a conventional flap in M3M extractions has any impact on the periodontal situation [37]. However, a rigid sealing of the site appears to be important in preventing wound healing disturbance and food impaction in the socket area, which is difficult to access when intentionally left open. Flapless surgery and—to certain degree—miniflap surgery as performed in the present case series have the advantage of preserving the periosteal protection of the lateral bone surface, which prevents any unintended lateral bone resorption. However, it cannot be excluded that the occlusal exposure provokes part of the observed bone reduction, which currently appears to be inevitable. However, it has the disadvantage of leaving an occlusal open surgical site for partially erupted teeth. The application of in situ hardening bone filling material has the ability to protect the M3M alveolus in the critical early phase of blood clot formation and subsequently serves as a scaffold for bone regeneration. The loss of particles at the surface does not interfere with wound healing. In our study it could be observed that a large majority of the space taken originally by the tooth is filled with mineralized tissue. Thus, a formation of deep soft tissue pockets is prevented by the underlying bone filler in the critical early phase of healing. Pericoronal cysts can thus be filled completely without raising a flap to prevent invagination of gingival tissue.

## 5. Conclusion

The present report gives evidence that a combination of inward fragmentation and the use of an in situ hardening bone filler is able to act synergistically. This minimally invasive concept for M3M surgery leads to a preservation of 90% of lingual and buccal alveolar bone height and prevents the formation of central alveolar bone defects.

## Figures and Tables

**Table 1:** Postoperative symptoms.

	Day 2		Day 7	
	Swelling Level (0-3)	Pain Level (0-10)	Swelling Level (0-3)	Pain Level (0-10)
Mean	1.00	2.31	0.23*∗*	1.08*∗*
Min	0	1	0	0
Max	2	6	1	2
Std. Dev	0.57	1.49	0.44	0.76

**Table 2:** Bone height at M3M sites: preoperative (Pre-BH) and 3 months postoperative mandibular bone height (Post-BH) at the M3M site 6 mm distal to the second molar distal limit.

Distance of the mandibular base to the occlusal margin	Pre-BH (mm)	Post-BH (mm)
Lingual (ML)	26.69	25.15
Central (MC)	26.24	23.33
Buccal (MB)	26.68	24.60

## Data Availability

The data used to support the findings of this study are included within the article.

## Abbreviations

3D: Three dimensions
BH: Bone height
B: Buccal
C: Central
CBCT: Cone-beam computed tomography
DFDBA: Demineralized freeze-dried bone allograft
GTR: Guided tissue regeneration
IAN: Inferior alveolar nerve
IFT: Inward fragmentation technique
L: Lingual
M3M: Mandibular third molars
M2M: Mandibular second molars
M: Mandibular base
PRF: Platelet rich fibrin
Pre-BH: Preoperative bone height
RCT: Randomized controlled trials
SD: Standard deviation
VAS: Visual analog scale
TCP: Tricalcium phosphate.
